# Waves of Change: Brain Sensitivity to Differential, not Absolute, Stimulus Intensity is Conserved Across Humans and Rats

**DOI:** 10.1093/cercor/bhaa267

**Published:** 2020-10-07

**Authors:** R Somervail, F Zhang, G Novembre, R J Bufacchi, Y Guo, M Crepaldi, L Hu, G D Iannetti

**Affiliations:** Department of Neuroscience, Physiology and Pharmacology, University College London, London, WC1E 6BT, UK; Neuroscience and Behaviour Laboratory, Istituto Italiano di Tecnologia, 00161 Rome, Italy; CAS Key Laboratory of Mental Health, Institute of Psychology, 100101 Beijing, China; Department of Psychology, University of Chinese Academy of Sciences, 100049 Beijing, China; Neuroscience and Behaviour Laboratory, Istituto Italiano di Tecnologia, 00161 Rome, Italy; Neuroscience and Behaviour Laboratory, Istituto Italiano di Tecnologia, 00161 Rome, Italy; Neuroscience and Behaviour Laboratory, Istituto Italiano di Tecnologia, 00161 Rome, Italy; Electronic Design Laboratory, Istituto Italiano di Tecnologia, 16152 Genova, Italy; CAS Key Laboratory of Mental Health, Institute of Psychology, 100101 Beijing, China; Department of Psychology, University of Chinese Academy of Sciences, 100049 Beijing, China; Department of Neuroscience, Physiology and Pharmacology, University College London, London, WC1E 6BT, UK; Neuroscience and Behaviour Laboratory, Istituto Italiano di Tecnologia, 00161 Rome, Italy

**Keywords:** electrocorticography (ECoG), electroencephalography (EEG), behavioral relevance, multispecies investigation, saliency-detection

## Abstract

Living in rapidly changing environments has shaped the mammalian brain toward high sensitivity to abrupt and intense sensory events—often signaling threats or affordances requiring swift reactions. Unsurprisingly, such events elicit a widespread electrocortical response (the vertex potential, VP), likely related to the preparation of appropriate behavioral reactions. Although the VP magnitude is largely determined by stimulus intensity, the relative contribution of the differential and absolute components of intensity remains unknown. Here, we dissociated the effects of these two components. We systematically varied the size of abrupt intensity increases embedded within continuous stimulation at different absolute intensities, while recording brain activity in humans (with scalp electroencephalography) and rats (with epidural electrocorticography). We obtained three main results. 1) VP magnitude largely depends on differential, and not absolute, stimulus intensity. This result held true, 2) for both auditory and somatosensory stimuli, indicating that sensitivity to differential intensity is supramodal, and 3) in both humans and rats, suggesting that sensitivity to abrupt intensity differentials is phylogenetically well-conserved. Altogether, the current results show that these large electrocortical responses are most sensitive to the detection of sensory changes that more likely signal the sudden appearance of novel objects or events in the environment.

## Introduction

Animals face a dynamic and potentially dangerous environment. The ability to detect abrupt and unexpected sensory events requiring immediate behavioral responses is key to survival. It is, therefore, no surprise that abrupt sensory stimuli elicit one of the largest and most widespread transient electrocortical responses detectable using scalp or epidural recordings, likely related to the preparation of appropriate behavioral reactions (Moayedi et al. 2015; Novembre et al. 2018). This response has been described in a number of animals including rats (Knight et al. 1985; Hu et al. 2015; Guo et al. 2016; Hu and Iannetti 2019), monkeys (Kulics 1982; Gardner et al. 1984; Neville and Foote 1984; Pineda et al. 1989; Beydoun et al. 1997), and humans (Bancaud et al. 1953; Walter 1964; Mouraux and Iannetti 2009).

In the human electroencephalogram (EEG), this response is dominated by a large and widespread negative–positive (N–P) wave maximal at the scalp vertex, often referred to as the vertex wave or vertex potential (VP) (Bancaud et al. 1953; Walter 1964; Novembre et al. 2018), which overlaps with a number of smaller and more localized components arising from activation of primary sensory cortices (Mouraux and Iannetti 2009; Valentini et al. 2012; Hu, Valentini et al. 2014). The VP and its underlying neural network can be recruited by stimuli belonging to different sensory modalities, provided that they are sufficiently salient (Bancaud et al. 1953; Mouraux and Iannetti 2009; Liang et al. 2010). We recently described a basic physiological mechanism that tightly couples VPs with a complex modulation of motor output, suggesting that VPs are unavoidably entwined with behavioral reactions (Novembre et al. 2018). An equivalent response with electrophysiological features and functional properties similar to the human VP can be recorded in freely behaving rats using, for example, electrocorticography (ECoG) (Hu et al. 2015; Xia et al. 2016).

It is well-established that stimulus intensity largely determines VP magnitude (Davis and Zerlin 1966; Davis et al. 1968; Schweitzer and Tepas 1974; Bromm and Treede 1991; Beydoun et al. 1993; Iannetti et al. 2005, 2008; Huang et al. 2013; Hu, Cai et al. 2014). However, what is usually labeled “stimulus intensity” reflects two distinct components that are often conflated (e.g., in all references above): differential and absolute intensity. “Differential intensity” refers to the difference between the baseline and target intensity. In contrast, “absolute intensity” can be formalized as the baseline from which an intensity increase takes place, or the target at which the intensity increase arrives, or any other absolute measure in between the baseline and the target (in our experiments, we formalized absolute intensity as the target intensity see Materials and Methods for details): for example, a difference of 2 units could occur at a low absolute level (from 2 to 4) or a high absolute level (from 9 to 11).

To the best of our knowledge, the relative importance of these two components in eliciting a VP has not been dissected. Indeed, VPs are usually elicited by impulse stimulation, in which stimulus intensity rises from zero to the desired target value, plateaus for a short time, and then drops back to zero (Davis and Zerlin 1966; Davis et al. 1968; Schweitzer and Tepas 1974; Bromm and Treede 1991; Beydoun et al. 1993; Iannetti et al. 2005, 2008; Huang et al. 2013). Obviously, with this type of stimuli, differential and absolute intensity covary, and are therefore indistinguishable.

**Figure 1:**
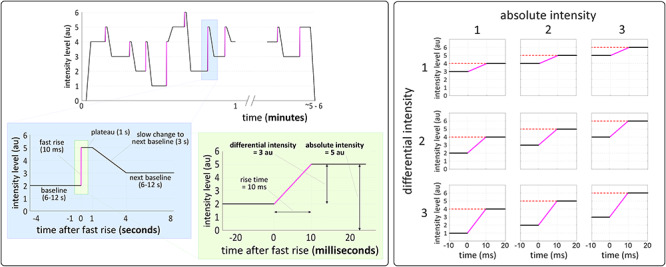
Stimulation profile and experimental design. Left panel: stimulation profile of a typical block of all three experiments. Stimulus intensity abruptly increased from a baseline level to a target level in 10 ms, plateaued for 1 s and then slowly increased or decreased to the next baseline in 3 s. Right panel: differential and absolute intensity were modulated using a 3 × 3 factorial design. Each abrupt increase occurred between different baseline and target levels, thus yielding 9 conditions varying in their differential intensity (the difference between baseline and target) and absolute intensity (here defined as the target intensity reached by the increase).

To this end, we conducted three experiments in humans and rats using a paradigm that allowed us to clearly dissociate differential and absolute stimulus intensity. We delivered continuous auditory or somatosensory stimuli with embedded abrupt intensity increases of different sizes occurring at different absolute levels, using a 3 × 3 factorial design (Fig. 1). In Experiments 1 and 2, we recorded scalp EEG from 36 human participants while delivering auditory and vibrotactile stimuli respectively. In Experiment 3, we recorded activity directly from the brain surface (EcoG) of 5 rats while delivering auditory stimuli.

We hypothesized that differential intensity would be the main factor determining VP magnitude. While it is well-known that a higher sensitivity to sensory differentials than to absolute intensity is a common property of peripheral receptors (e.g., muscle stretch receptors; Hulliger et al. 1977; Hunt and Wilkinson 1980; Blum et al. 2017), it remains unknown whether widespread event-related brain potentials also show similar sensitivity. Importantly, such brain potentials and their underlying neural processes serve higher-level functions than peripheral receptors, and therefore their sensitivity to different environmental features is more complex, and crucially depends on those functions (Ronga et al. 2013). For example, we have previously demonstrated that the VP reflects the salience and behavioral relevance of abrupt environmental events (Iannetti et al. 2008; Valentini et al. 2011; Ronga et al. 2013; Moayedi et al. 2016; Novembre et al. 2018). Given that differential intensity largely contributes to salience (see discussion), we expected it to strongly modulate the VP magnitude.

## Materials and Methods

### Experiments 1 and 2

#### Human participants

A total of 36 healthy human participants took part in Experiments 1 and 2 (*N* = 18 unique participants in each experiment). In Experiment 1 (11 female, mean age 27, age-range 21–46), EEG data were collected at UCL, London, UK. In Experiment 2 (10 female, mean age 34, age-range 24–71), EEG data were collected at IIT, Rome, Italy. All participants gave written informed consent before taking part in the study. All procedures were approved by the respective local ethical committees.

#### Sensory stimuli

In Experiment 1, participants received tonic auditory stimuli. Auditory stimuli were 600 Hz pure tones delivered binaurally through pneumatic insert-earphones (Etymotic ER-3C 10 Ohm). Auditory stimulation was controlled using Presentation® (Neurobehavioral Systems). In Experiment 2, participants received tonic vibrotactile stimuli. Vibrotactile stimuli were delivered through a stimulator attached to the participants’ left index finger (Z7A-series DC motor, Jinlong Machinery & Electronics, China), while participants sat with the stimulated hand resting on their lap with the palm facing upwards. The vibrotactile stimulator was driven by a Texas Instruments DRV2605 haptic driver with a Real Time Playback interface connected to an ATSAMD21 Cortex-M0 microcontroller. The processor receives set points from a host PC through a USB-emulated Universal Asynchronous Receiver-Transmitter interface. The Cortex-M0 runs a low-level firmware that asynchronously decodes an amplitude set point received from the PC (from 0 to 127) and sets the haptic driver accordingly. Vibrotactile stimuli were controlled at a high level using MATLAB (MathWorks) and the Psychophysics Toolbox (Brainard 1997). In Experiment 2, white noise was continuously delivered through the same earphones used in Experiment 1, to prevent participants from hearing the vibrotactile stimulator. No participant reported hearing the vibrotactile stimulator while white noise was played.

#### Experimental design

In both experiments, abrupt (10 ms long) increases of stimulus intensity were embedded within a tonic stimulation (Fig. 1, left panel). These increases were of three levels of differential intensity and reached one of three levels of absolute intensity (Fig. 1, right panel). This resulted in a 3 × 3 factorial design, with 9 conditions in total. The onsets of the intensity increases were subsequently used for EEG time-lock analysis.

Each experiment consisted of 8 blocks, with 27 intensity increases per block (3 per condition), yielding 216 increases in total (24 of each condition). Figure 1 (left panel) shows the stimulation profile of a representative block: before the first stimulus, the baseline level was set by slowly rising the intensity level from zero (3 s). After each abrupt increase, stimulus intensity remained at the target level for 1 s. After this plateau, the intensity level slowly increased or decreased to reach the baseline of the next trial. The slow increase or decrease lasted 3 s, to avoid eliciting another VP. After the last stimulus of each block, the intensity slowly decreased to zero (3 s). The mean interval between two consecutive stimulus increases (i.e., between two trials) was 13 s (10–16 s). The 9 conditions were presented in random order, with the constraint that no more than 2 trials of the same condition were presented consecutively. Participants were allowed to rest for approximately 2 min between two consecutive blocks.

#### Preliminary definition of stimulus intensity levels

The stimulation paradigm entailed 6 equally spaced intensity levels (these levels were equally spaced with respect to perceived intensity, rather than stimulus energy). These 6 levels were determined in a preliminary psychophysical experiment conducted in 5 participants, separately for Experiments 1 and 2, using the following procedure. Levels were adjusted to ensure that all increases of intensity with a particular differential were perceived as being comparable, regardless of absolute intensity (e.g., to ensure that the perceived differential from level 2 to 4 and from level 3 to 5 was similar). Participants were asked to manually adjust the intensity levels using a keyboard and a custom graphical interface. At the beginning of this psychophysical experiment, the lowest level was set at the minimal clearly detectable intensity, and the highest level was set at the minimal comfortable intensity. The levels chosen by each participant to achieve a similar perception of differential intensity were finally averaged across participants. These average levels were used for all participants in subsequent EEG experiments. We also performed an additional control experiment with auditory stimuli, in which the preliminary psychophysical intensity level definition was performed separately by each participant before taking part in the main experiment (see details in the legend of Fig. S1). This control experiment examined whether interparticipant variability in the stimulus–perception relationship affected our results, and produced very similar results to Experiment 1 (see Fig. S1).

#### E‌EG recording and preprocessing

Brain activity was recorded using a 29-channel wireless EEG system (Quick-30, Cognionics, USA; 500 Hz sampling rate). During acquisition, participants were required to keep their gaze on a fixation cross (4 × 4 cm) placed centrally in front of them, at approximately 30° below eye-level. EEG signals were preprocessed and analyzed using MATLAB (version 2018a, MathWorks) and Fieldtrip (Oostenveld et al. 2011). Continuous EEG data were first band-pass filtered between 0.5 and 30 Hz (Butterworth). Data were then segmented into epochs using a time-window of ±2 s from stimulus onset (epoch duration = 4 s). Artifacts due to eye blinks or eye movements were removed using a validated method based on independent components analysis (Jung et al. 2000). Within each epoch, any electrode with amplitude values exceeding ±100 μV in no more than 3 electrodes was interpolated by averaging neighboring electrodes; if more than 3 electrodes required interpolation, the epoch was rejected. Remaining epochs were baseline corrected between 200 ms prestimulus and stimulus onset, and then visually inspected for remaining artifacts to be rejected. The average number of rejected epochs per subject was 22 ± 14 SD (i.e., approximately 10% of the total number of epochs) in Experiment 1 and 10 ± 8 (i.e., approximately 5% of the total number of epochs) in Experiment 2. The number of rejected epochs was not different across experimental conditions in Experiment 1 (1-way ANOVA: *P* = 0.99), Experiment 2 (*P* = 0.29), and the control experiment (*P* = 0.98). Finally, epochs of the same condition were averaged, yielding 9 average waveforms for each participant. VP peaks were also extracted from the across-trial average of each participant and condition, using the following procedure. We first calculated the average response of each participant across all stimulus conditions. We then identified, on this average response, two-time windows, each centered on the N and the P wave peaks. We used these time windows to extract separately, from each condition waveform and for each subject, the amplitude and latency of each of the two peaks. The mean peak latencies across conditions and participants were as follows. N wave: 113 ± 13 ms; P wave 212 ± 27 ms (Experiment 1; auditory stimulation); N wave: 164 ± 24 ms; P wave: 261 ± 42 ms (Experiment 2; somatosensory).

#### Statistical analysis

Single-subject average waveforms of each condition were analyzed using a linear mixed-effect (LME) model (MATLAB, Statistics and Machine Learning Toolbox) at each timepoint and electrode, with “differential intensity” and “absolute intensity” as fixed effects and “participant” as a random effect. To correct for multiple comparisons, we used a cluster permutation test with 2000 permutations (Maris and Oostenveld 2007; Phipson and Smyth 2010) across all channels and timepoints within the time window −200 ms to +600 ms. In addition, to ascertain whether the LME results obtained using the point-by-point analysis were consequent to a modulation of response latencies, we analyzed the peak latency values extracted from the average waveform of each subject and condition using an LME model with the same experimental factors described above. To test for an interaction between the factors “differential intensity” and “absolute intensity,” we also performed a two-way repeated-measures ANOVA with false discovery rate (FDR) correction for all three EEG experiments. These tests showed no evidence for interaction effects (see Fig. S2).

### Experiment 3

#### Animals & surgical procedure

The experiment was conducted on 5 adult male Sprague Dawley rats weighing 300–400 g at the Chinese Academy of Sciences, Beijing, China. Rats were fed ad libitum with water and food and were housed in separate cages under temperature- and humidity-controlled conditions. They were kept in a 12 h day/night cycle (lights on from 19:00–7:00). All experimental procedures adhered to local guidelines for animal experimentation and were approved by the local ethics committee. Surgical procedures and electrode positioning are detailed elsewhere (Xia et al. 2016; Jin et al. 2018; Zhang et al. 2019). Following surgery, rats were kept in individual cages for at least 7 days before the collection of ECoG data.

#### Sensory stimuli

Auditory stimulation was an 8000 Hz pure tone delivered from a loudspeaker placed below the cage (but not in contact with the cage floor). The difference in frequency of stimulation between the human and animal experiments reflects the between-species difference in auditory frequency sensitivity (Jamison 1951; Hess 2015). Stimuli were controlled using MATLAB (MathWorks) and the Psychophysics Toolbox (Brainard 1997). As in the human experiments, auditory stimuli were delivered at 6 intensity levels, equally spaced in terms of perceived intensity. Unlike in the human experiment, these levels were defined using the rat power-law relationship between sound pressure level and perceived intensity (Pierrel-Sorrentino and Raslear 1980; Raslear 1989). A similar power-law relationship was observed when relating sound pressure levels and perceived intensity reported by the human participants in the preliminary definition of stimulus intensity levels.

#### Experimental design

Experimental design was identical to Experiments 1 and 2, with the exception that the baseline periods had variable duration, given that abrupt increases of stimulus intensity had to be delivered manually by the experimenter after at least 6 s of baseline, when the animal was calm and not moving. As a result, the duration of the baseline period ranged between 11 and 131 s (median = 16.3 s). Each rat received 27 abrupt increases in each of 12 blocks, yielding 324 intensity increases in total (36 per condition).

#### ECoG recording & preprocessing

Cortical activity was recorded using a 14-channel wireless amplifier system (Multi Channel System MCS Gmbh, Germany; 2000 Hz sampling rate). During recording, rats were placed into a plastic chamber (length × width × height: 30 × 30 × 30 cm^3^), within which they could move freely. Before the data collection, rats were placed in the same plastic cage for at least 4 slots of 2 h each, to familiarize them with the recording environment. ECoG signals were processed using the EEGLAB toolbox (Delorme and Makeig 2004). Raw ECoG data were downsampled to 1000 Hz, bandpass filtered from 1 Hz to 100 Hz, and finally segmented into epochs using a time-window ranging from −200 to +500 ms. Epochs with amplitudes exceeding ±500 μV were excluded from further analysis. The average number of rejected epochs per rat was 9 ± 5 SD (i.e., 3% of the total number of epochs). The number of rejected epochs was not different across experimental conditions (1-way ANOVA: *P* = 0.31).

#### Statistical analysis

Data collected in Experiment 3 were analyzed using the same LME and cluster permutation testing approach used in Experiments 1 and 2. However, because the number of animals tested in Experiment 3 (*n* = 5) was lower than the number of humans tested in Experiment 1 and 2 (*n* = 18 each), we entered single epochs instead of single-subject averages into the model, to make statistical power comparable across distinct datasets.

## Results

### Experiment 1: Auditory Stimulation in Humans

#### E‌EG waveform & topographies

**Figure 2:**
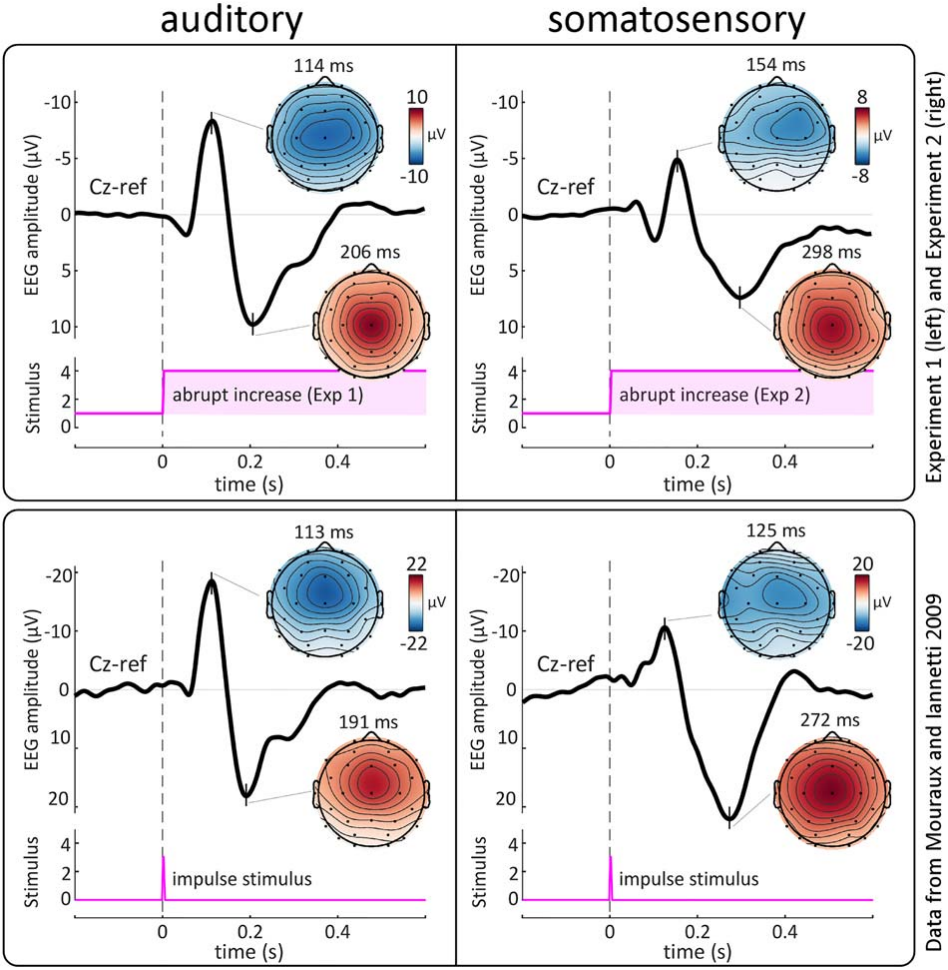
Experiments 1 and 2: abrupt intensity increases embedded in ongoing stimuli elicit VPs remarkably similar to those commonly evoked by impulse stimuli. Top panel: grand average EEG responses elicited by abrupt increases of intensity of continuous auditory (left) and somatosensory (vibrotactile, right) stimulation. Data from Experiments 1 and 2. Bottom panel: grand average EEG responses to auditory (left) and somatosensory (electrical, right) impulse stimuli. Data from Mouraux and Iannetti (2009). In both panels the EEG amplitude timecourse at Cz is shown in black. Vertical dashed lines indicate stimulus onset. Pink plots show stimulus profiles. Scalp topographies are shown at the peak latency of the negative and positive VPs. Note how abrupt intensity increases embedded in ongoing stimuli elicit VPs (top panels) remarkably similar to those elicited by commonly used impulse stimuli (bottom panels). Note also the longer latencies of the N and P waves elicited by vibrotactile stimuli (top panel, right) compared to electrical stimuli (bottom panel, right), given that electrical stimulation bypasses the mechanoreceptors and directly activates axons of Aβ afferents.

**Figure 3:**
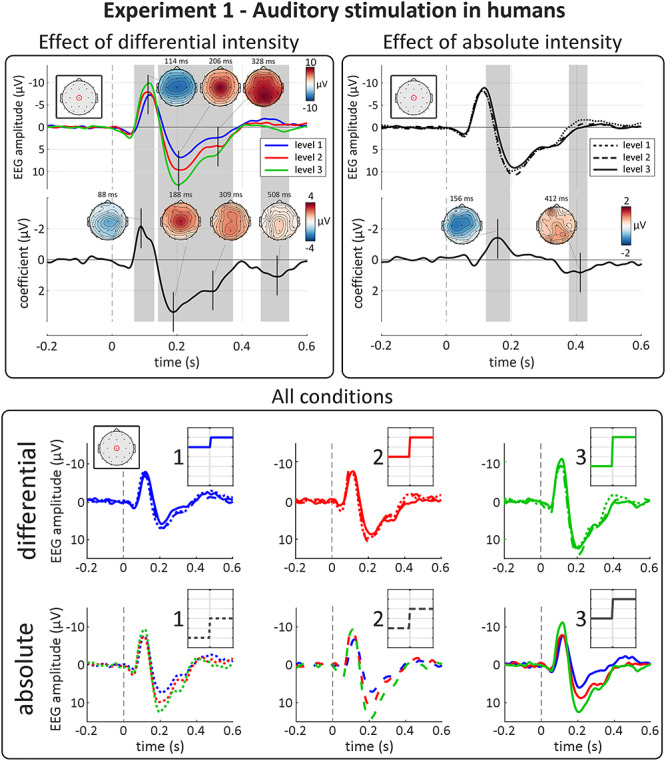
Experiment 1: auditory-evoked VPs are highly sensitive to differential, not absolute, intensity. Top panels show the results of point-by-point LME analysis. Top plots show group-level average waveforms at Cz for each of the three levels of differential (left panel) and absolute intensity (right panel). Bottom plots show the LME model coefficient timecourse for each factor. Gray areas show significant clusters after permutation testing. Vertical dashed lines indicate stimulus onset. The amplitude of both negative and positive waves were strongly modulated by the factor “differential intensity.” The peak topographies of these effects correspond well to those of the EEG response. The apparent amplitude modulation at the inflection point of the VP by absolute intensity was consequent to a small latency shift (with higher absolute intensity resulting in longer-latency responses, see Results) rather than a modulation of magnitude per se. Bottom panel show group-level average waveforms at Cz, for each condition. Each row shows all 9 conditions of the experiment. Insets show schematic stimulus profiles, for each condition. Note the effect of differential, but not absolute intensity on both the negative and positive VPs.

In Experiment 1, we recorded the human EEG responses to abrupt increases the intensity of an ongoing auditory stimulus. Figure 2 (top-left panel) shows the grand average EEG response. Abrupt increases of stimulus intensity elicited a large N–P complex, peaking at approximately 110 and 210 ms, respectively. Both the N and P waves had maximal amplitude at the vertex, but while the N topography extended more toward the temporal leads, the P topography decayed similarly in all directions away from the vertex (Fig. 2, top-left panel). A smaller positive deflection peaking at approximately 330 ms followed the main P wave. This later positive peak had a more posterior topography with a maximum over Pz, possibly reflecting a P3b response (Polich, 2007; Fig. 3, top-left panel). Overall, the waveform shape and topography of the N and P waves were very similar to the VPs elicited by transient impulse auditory stimuli (Fig. 2, bottom-left panel; Picton and Hillyard, 1974; Thomson et al. 2009; Valentini et al. 2011).

#### Effect of “differential intensity”

**Figure 4:**
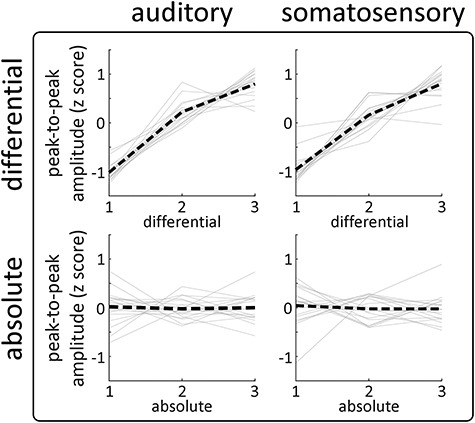
Experiments 1 and 2: the effect of differential and absolute intensity on the VPs is consistent across modalities and participants. Each graph shows the peak-to-peak amplitude of the VPs for each participant (gray lines), together with the group-level average (black line) for each experimental factor (rows) and sensory modality (columns). Note the strong positive relationship between “differential intensity” and response amplitude in both modalities, remarkably consistent across participants. There was no consistent effect of “absolute intensity” on response amplitude.

Differential intensity strongly modulated the magnitude of both the N and P components of the VP (Fig. 3, top-left panel). The left column of Figure 4 shows the VP peak-to-peak amplitude extracted from each subject for the three levels of differential and absolute intensity. The modulation of VP magnitude by differential intensity was highly consistent across participants, with larger differentials eliciting larger responses. This modulation was similar at each of the three levels of absolute intensity (Fig. 3, bottom panel). These observations were substantiated by LME modeling and cluster-permutation testing, which showed strong evidence that the factor “differential intensity” affected the amplitude of the signal in two-time windows across many electrodes: a negative cluster (*P* = 0.0005 at 2000 permutations) at 70–130 ms, and a double-peaked positive cluster (*P* = 0.0005) at 140–370 ms. Note that with permutation testing the p value is calculated according to the formula p = b+1/m+1, where *m* is the number of performed permutations, and *b* is the number of permutations giving a larger test statistic than the actual test statistic. Therefore, p = 0.0005 is the smallest possible p value, obtained when none of the 2000 permutations had a test statistic larger than the actual one (Phipson and Smyth 2010). The two peaks of maximal modulations (at 88 and 190 ms, respectively) had both latency and topography similar to the peaks of the VP (Fig. 3, top-left panel). These modulations were large: LME estimated the amplitude of the negative/positive peaks to increase by −2.2/3.4 μV at each subsequent level of differential intensity (i.e., 26% and 34% of the respective grand average amplitudes). We also found that differential intensity modulated the EEG amplitude in a later time window, well after the end of the VP (at 460–540 ms; *P* = 0.0015; peak coefficient = 1.1 μV).

#### Effect of “absolute intensity”

In contrast with the strong effects of differential intensity, we observed no clear modulation of VP magnitude by absolute stimulus intensity (Figs 3 and 4). LME confirmed that the factor “absolute intensity” did not affect the overall magnitude of the N and P waves (Fig. 3, top-right panel), although there was an effect within a cluster around the inflection point between the N and P components of the VP (*P* = 0.0005). This cluster most likely reflected a latency difference when the VP was elicited by stimuli of different absolute intensity—an interpretation supported by the LME analysis performed on the individually extracted peak latencies, which showed evidence that “absolute intensity” affected the latency of both the N (*P* = 0.002) and P (*P* = 0.048) waves. Finally, point-by-point LME revealed that “absolute intensity” had a small effect in a late positive cluster well after the VP, at 370–430 ms (*P* = 0.0015; peak coefficient = 0.9 μV), with a slightly posterior and right-lateralised peak topography.

### Experiment 2: Somatosensory Stimulation in Humans

#### E‌EG waveform & topographies

In Experiment 2, abrupt increases in the intensity of ongoing somatosensory stimulation elicited a large N–P complex, peaking at approximately 150 and 300 ms (Fig. 2, top-right panel). The scalp distribution of the P wave was clearly maximal at the vertex, whereas that of the N wave was slightly more frontal and contralateral to the stimulated hand, due to the overlap with smaller somatosensory-specific components (Treede et al. 1988; Valentini et al. 2012; Hu, Valentini et al. 2014). Overall, the shape and topography of the N and P waves were similar to the VPs elicited by transient impulse somatosensory stimuli (Fig. 2, bottom-right panel; Valentini et al. 2012).

#### Effect of “differential intensity”

**Figure 5:**
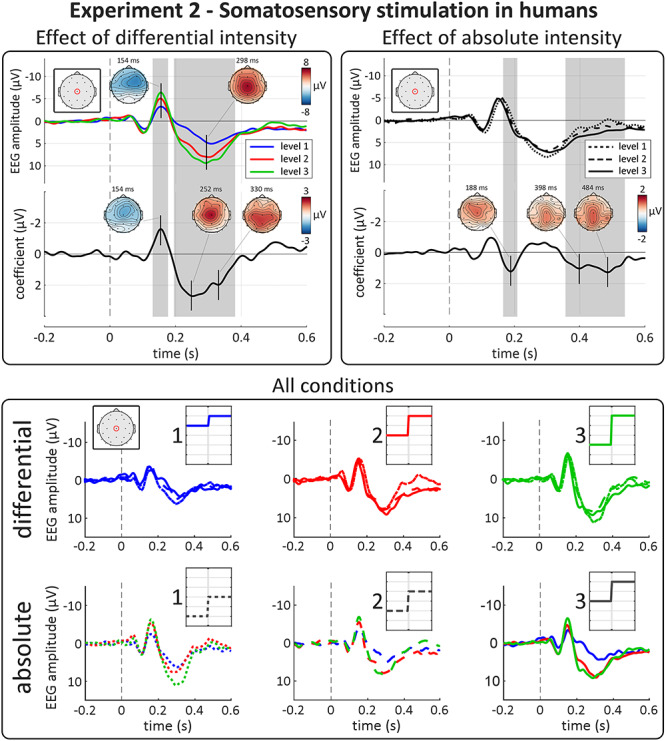
Experiment 2: somatosensory-evoked VPs are highly sensitive to differential, not absolute, intensity. Top panels show results of point-by-point LME analysis. Top plots show group-level average waveforms at Cz for each of the three levels of differential (left panel) and absolute intensity (right panel). Bottom plots show the LME model coefficient timecourse for each factor. Gray areas show significant clusters after permutation testing. Vertical dashed lines indicate stimulus onset. The amplitude of both negative and positive waves were strongly modulated by the factor “differential intensity.” As expected, the peak topographies of these effects were maximal at the vertex, suggesting that the slightly unusual topography of the N wave in the EEG average reflects the superimposition of the VP and another component, perhaps generated by the primary somatosensory cortex contralateral to the stimulated hand (Valentini et al. 2012; Hu, Valentini et al. 2014). The apparent amplitude modulation at the inflection point of the VP by absolute intensity was consequent to a small latency shift (with higher absolute intensity resulting in shorter-latency responses, see Results section) rather than a modulation of magnitude per se. There was again a late positive cluster, well after the VP, modulated by “absolute intensity.” Bottom panel shows group-level average waveforms at Cz, for each condition. Each row shows all 9 conditions of the experiment. Insets show schematic stimulus profiles, for each condition. Note the effect of differential, but not absolute, intensity on both the negative and positive VPs.

As in Experiment 1, differential intensity strongly modulated the magnitude of both the N and P waves (Fig. 5, top-left panel). The right column of Figure 4 shows the VP peak-to-peak amplitude extracted from each subject for the three levels of differential and absolute intensity. Again, the modulation of VP magnitude by differential intensity was highly consistent across participants, with larger differentials eliciting larger responses. This modulation was similar at each of the three levels of absolute intensity (Fig. 5, bottom panel). These observations were substantiated by LME modeling and cluster-permutation testing, which showed strong evidence that the factor “differential intensity” affected the amplitude of the signal in two-time windows across many electrodes: a negative cluster (*P* = 0.0005) at 130–180 ms, and a double-peaked positive cluster (*P* = 0.0005) at 210–380 ms. The two peaks of maximal modulation had centrally distributed topographies indicating that the effects were driven by the VP, rather than the modality-specific components that overlap with the N wave (Fig. 5, top-left panel; Treede et al. 1988; Valentini et al. 2012; Hu, Valentini et al. 2014). As in Experiment 1, these modulations were large: LME estimated the amplitude of the negative/positive peaks to increase by −1.6/2.7 μV at each subsequent level of differential intensity (i.e., 33% and 36% of the respective grand average amplitudes).

#### Effect of “absolute intensity”

As in Experiment 1, we observed no clear modulation of VP magnitude by absolute stimulus intensity (Figs 4 and 5). LME confirmed that the factor “absolute intensity” did not affect the overall magnitude of the N and P waves (Fig. 5, top-right panel), although there was an effect within a cluster around the inflection point between the N and P waves (*P* = 0.0005). As in Experiment 1, this cluster likely reflected a latency difference when the VP was elicited by stimuli of different absolute intensity (although in the opposite direction to Experiment 1) instead of a true modulation of the wave magnitude—an interpretation supported by the LME analysis performed on the individually extracted peak latencies, which showed evidence that “absolute intensity” affected the latency of both the N (*P* = 5e^−5^) and P (*P* = 0.02) waves. Finally, LME revealed that “absolute intensity” had a small effect in a late positive cluster well after the VP, at 390–540 ms (*P* = 0.0005; peak coefficient = 1.3 μV), with a central and slightly posterior topography.

### Experiment 3: Auditory Stimulation in Rats

#### ECoG waveforms & topographies

**Figure 6:**
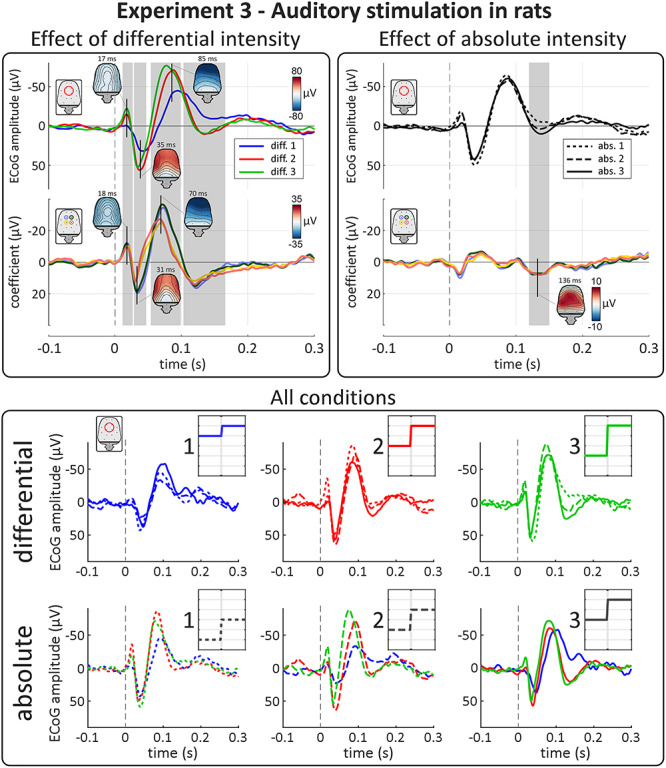
Experiment 3: like human ERPs, auditory ERPs in rats are highly sensitive to differential, not absolute, intensity. Top panels show results of point-by-point LME analysis. Top plots show group-level waveforms of the average of four summary electrodes for each of the three levels of differential (left panel) and absolute intensity (right panel). Bottom plots show the model coefficient timecourse for each factor, separately for each electrode used in the averages. Gray areas show significant clusters after permutation testing. Vertical dashed lines indicate stimulus onset. All three main components of the response were strongly modulated by the factor “differential intensity,” with effect topographies matching those of the peaks of the ECoG response. In contrast, the main three components were not modulated at all by “absolute intensity.” There were some late effects of “absolute intensity” and “differential intensity” after the third component of the response, at ~ 121–136 ms. Bottom panel shows group-level average waveforms of the average of four summary electrodes for each condition. Each row shows all 9 conditions of the experiment. Insets show schematic stimulus profiles for each condition. Note the effect of differential, but not absolute intensity on the main three components of the ECoG response.

In Experiment 3, we recorded ECoG from rats, while delivering auditory stimuli using the same procedure as in human Experiment 1. Abrupt increases of stimulus intensity elicited large deflections in the time domain ECoG signal (Fig. 6). These consisted of three components with expectedly shorter latencies than their human counterpart (Hu et al. 2015): 1) a fronto-lateral negativity peaking at 17 ms, 2) a fronto-lateral positivity peaking at 35 ms, and 3) a frontal negativity peaking at 85 ms. The shape and topography of these components correspond well to previously reported ECoG responses to transient impulse auditory stimuli (Knight et al. 1985; Hu et al. 2015; Guo et al. 2016).

#### Effect of “differential intensity”

Similar to what we observed in the human experiments, all main components of the electrocortical response were strongly modulated by differential intensity (Fig. 6, top-left panel). LME showed strong evidence of three clusters in which the response magnitude was larger with larger differential intensity. These clusters had latencies similar to those of the ECoG response components: 1) a negative fronto-lateral cluster at 11–23 ms (*P* = 0.0105; peak coefficient = −15.8 μV) 2) a positive fronto-lateral cluster at 27–44 ms (*P* = 0.0130; peak coefficient = 26.2 μV), and 3) a negative frontal cluster at 45–98 ms (*P* = 0.0005; peak coefficient = −46.9 μV). We also observed an additional positive frontal cluster at 108–153 ms (*P* = 0.0005; peak coefficient = 20.1 μV), after the main three components. Note that these peak coefficients are calculated across all electrodes and are therefore not necessarily reflected in Fig. 6, which shows the coefficients timecourses from four summary electrodes.

#### Effect of “absolute intensity”

As in the human experiments, we observed no clear modulation of the amplitude of the three main components by absolute intensity (Fig. 6, top-right panel). Thus, the rat ECoG responses equivalent to the human VP were also sensitive only to differential and not absolute intensity. Again, LME revealed a late positive cluster at 121–150 ms (*P* = 0.0005; peak coefficient = 8.8 μV) whose amplitude was more positive for higher absolute intensity.

## Discussion

In this study conducted in humans and rats, we investigated the electrocortical responses elicited by sudden environmental changes embedded within tonic stimulation. Specifically, we exploited a paradigm that allows dissociating the effects of the differential and absolute components of stimulus intensity on response magnitude.

We obtained three main results: first, the VP magnitude is largely determined by differential intensity, independently of absolute intensity. This finding indicates that the widely-known effects of intensity on impulse-evoked VPs are driven by differential intensity. Second, this result was observed in the responses elicited by both auditory and somatosensory stimuli, indicating that sensitivity to differential intensity is supramodal. Third, the same effect was observed in both rats and humans, suggesting that sensitivity to abrupt intensity differentials is phylogenetically well-conserved.

### VPs are Sensitive to Differential, not Absolute, Intensity

**Figure 7:**
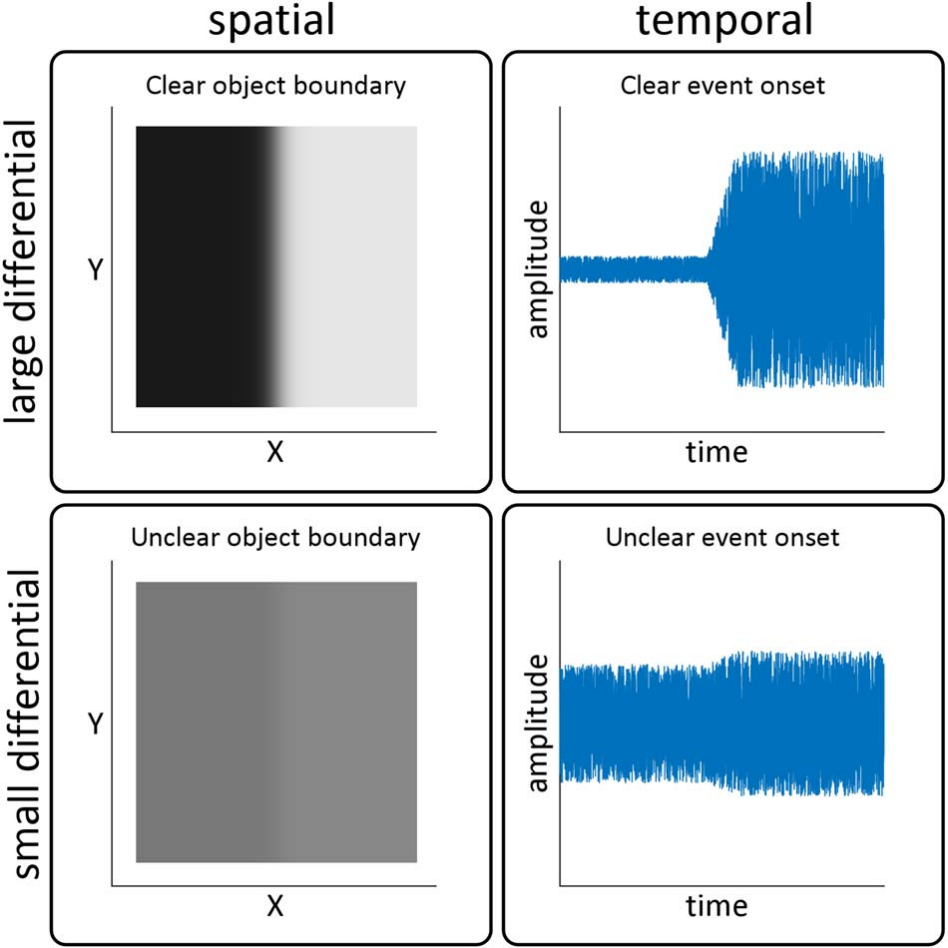
Abrupt increases of stimulus intensity are the temporal equivalent of spatial edges. Left column: representative plots of a spatial edge with large differential intensity (high contrast, top) and small differential intensity (low contrast, bottom). The large differential results in a sharper and more clearly defined edge, which identifies an object with higher certainty. Right column: abrupt increases of auditory intensity with large (top) and small (bottom) differentials. As in the visual domain, a larger differential results in a sharper, more clearly defined edge, albeit in time rather than in space. A sharper temporal edge identifies the occurrence of an event with higher certainty.

In all three experiments, the magnitude of the VPs evoked by the abrupt intensity increases was largely determined by differential, not absolute intensity, indicating that the differential intensity underlies the well-established effect of impulse stimulus intensity on VP magnitude (e.g., Davis and Zerlin, 1966; Davis et al. 1968; Schweitzer and Tepas, 1974; Bromm and Treede, 1991; Beydoun et al. 1993; Iannetti et al. 2005, 2008; Huang et al. 2013; Hu, Cai et al. 2014). Thus, the VP is highly sensitive to the degree to which an abrupt change stands out from the recent sensory input (i.e., from the baseline intensity). Interestingly, this description of differential intensity is reminiscent of a common definition of salience as “the degree to which a stimulus stands out from its surroundings” (Itti and Koch 2001; note that while the term “surroundings” is usually interpreted spatially, here it refers to the stimulus surroundings in time, i.e., the sensory input preceding the stimulus, see Fig. 7). Many other factors effectively modulating VP magnitude fall into this definition of salience: for example, the ratio of stimulus intensity to background noise (Baltzell and Billings 2014), or the degree to which an impulse stimulus stands out from the preceding sequence of stimuli. Indeed, the response habituation consequent to the repetition of the same stimulus at ~1 Hz (Iannetti et al. 2008; Wang et al. 2010; Herrmann et al. 2015) is reversed by behaviorally relevant changes of stimulus modality (Valentini et al. 2011), intensity (Ronga et al. 2013), pitch (Herrmann et al. 2015), and location in egocentric coordinates (Moayedi et al. 2016). Altogether, these results indicate that the VP is sensitive to the salience of environmental changes at several hierarchical levels and timescales. We discuss later how this sensitivity allows organisms to detect and respond appropriately to salient events in the environment.

### Sensitivity to Differential Intensity is Consistent Across Sensory Modalities

These results demonstrate that the sensitivity to differential intensity is present regardless of the sensory modality of the eliciting stimulus. This fits well with previous findings that VPs evoked by impulse stimuli of different modalities are similar in morphology, topography, and magnitude (provided that stimuli are saliency-matched; Mouraux and Iannetti 2009; Kilintari et al. 2018), that their habituation follows the same timecourse (Mancini et al. 2018), and that they share common supramodal generators (Mouraux and Iannetti 2009). Therefore, the results we observed here provide further evidence that the VP is a supramodal response that can be evoked by abrupt changes in the ongoing sensory input of any modality. It is worth highlighting that this supramodal response is often incorrectly assumed to reflect the processing of specific sensory modalities. A striking example is the widely-used label “acoustic-change complex” to refer to the EEG response elicited by changes in ongoing auditory stimuli (Martin and Boothroyd 1999, 2000). Although broadly accepted in the clinical arena, the implication that this response reflects auditory-specific processing is not supported by either present results or previous findings. The widespread use of a label implying an auditory-specific interpretation (e.g., Friesen and Tremblay, 2006; Hoppe et al. 2010; He et al. 2015; Mathew et al. 2017) could obstruct understanding of audiological pathophysiology and therefore misinform future clinical decisions. Similar misinterpretations affect the pain field, as we have discussed elsewhere (Hu and Iannetti 2016; Mouraux and Iannetti 2018).

### Which Neural Systems Underlie the Generation of VPs?

Information about the sensory environment is relayed by two main sensory pathways. Lemniscal pathways convey high-fidelity information in a given sensory modality to its primary sensory cortex, while extralemniscal pathways convey low-fidelity information to diffuse thalamic and cortical targets (Hu 2003). This anatomo-functional dichotomy is relevant to interpret our results: while the lemniscal system is sensitive to fine-grained stimulus features of one sensory modality, the extralemniscal system is sensitive to supramodal environmental changes, and rapidly habituates to repetitive stimulation (Calford and Aitkin 1983; Kraus et al. 1994; Edeline et al. 1999; Hu 2003; Komura et al. 2005; Anderson et al. 2009; Anderson and Linden 2011). There is clear evidence suggesting that the VP, which is a supramodal response that rapidly habituates to repetitive stimulation (Iannetti et al. 2008; Mouraux and Iannetti 2009), results from the activation of the extralemniscal system. Indeed, a crucial interventional study recording the electrocortical activity in free-behaving rats conclusively demonstrated that the VP elicited by abrupt auditory stimuli is largely unaffected by a bilateral ablation of the primary auditory cortex, whereas it strongly relies on a physiologically intact extralemniscal pathway (Simpson and Knight 1993). Additionally, dynamic causal modeling of human fMRI data demonstrated that salient sensory information reflecting abrupt stimuli is transmitted directly to non-sensory-specific regions such as insular and anterior cingulate cortex, bypassing primary sensory cortices (Liang et al. 2013). Together, these previous results suggest that the extralemniscal system is responsible for our present findings.

### Sensitivity to Differential Intensity: Lessons From the Natural World

What is the advantage of a neural system sensitive to differential intensity? A viable hypothesis is that the sensitivity to larger, more salient differentials allows organisms to respond to environmental changes on the basis of their relevance to immediate behavior. A large differential occurring in a short time acts as a sharper, more defined “edge” in the temporal dimension, analogously to a spatial edge in the visual domain (Fig. 7), and signals the occurrence of a new event or “object” with higher certainty (Chait et al. 2008). Indeed, animals face a dynamic sensory environment in which a sudden sensory event, whether the snap of a twig underfoot or a sudden ripple on the ocean surface, could signal the arrival of a predator or a critical opportunity to catch prey. Such situations would demand immediate action to successfully escape that predator or catch that prey—and therefore survive. Given the physiological cost of eliciting widespread brain activity and any subsequent behavioral response, prioritizing more certain environmental changes would allow the organism to minimize this cost as much as possible, without missing a potentially life-threatening event. The correct identification of a new object or event, therefore, has clear relevance to survival and wellbeing.

The striking similarity in sensitivity to differential intensity across humans and rats (Figs 3, 5 and 6) is interesting. Indeed, several aspects of sensory sensitivity differ dramatically across species: for example, the frequency of audible sounds in humans and rodents (Jamison 1951; Hess 2015) or the sampling rate of the visual system of humans and chickens (Zanker and Harris 2002; Lisney et al. 2011). These differences reflect different statistical properties of behaviorally relevant features in the habitats of the species (von Uexküll 1909; Hughes 2001). Our results suggest that the relevance of rapid increases of stimulus intensity is largely invariant in the habitats of both humans and rats and may be invariant across those of many other species. As a consequence, the neural system evolved to respond to these features is likely to be phylogenetically highly conserved across species.

## Supplementary Material

figure_S1_bhaa267

figure_S2_bhaa267

Somervail_et_al_supplementary_figure_legends_bhaa267

## Notes

We thank Cristina Salvatori for helping with data collection; and Marina Kilintari, Cédric Lenoir, Aoife Fitzpatrick, Atesh Koul, Prof. Roberto Caminiti, and Prof. Patrick Haggard for useful comments and discussions. We thank Claudio Lorini (IIT Electronic Design Laboratory, EDL) for the implementation of the vibrotactile stimulation system used in the experiments. *Conflict of Interest:* None declared.
