# Supplementary figures and images for: Waves of Change: Brain Sensitivity to Differential, not Absolute, Stimulus Intensity is Conserved Across Humans and Rats
Source: Cereb Cortex. 2020 Oct 7;31(2):949–60. doi: 10.1093/cercor/bhaa267 (PMC7786352; doi:10.1093/cercor/bhaa267)

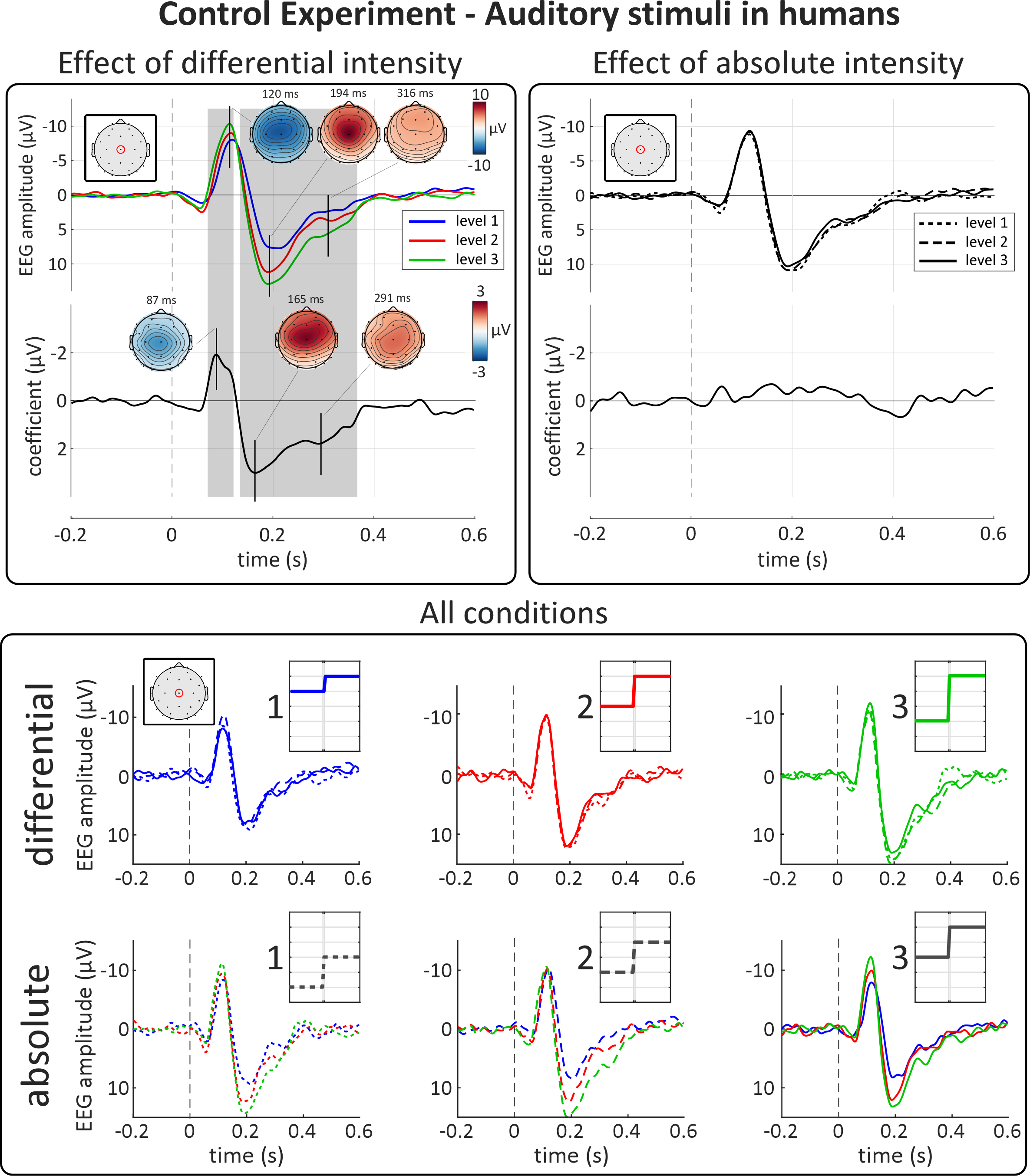

Supplement: figure_S1_bhaa267 [file figure_s1_bhaa267.png]

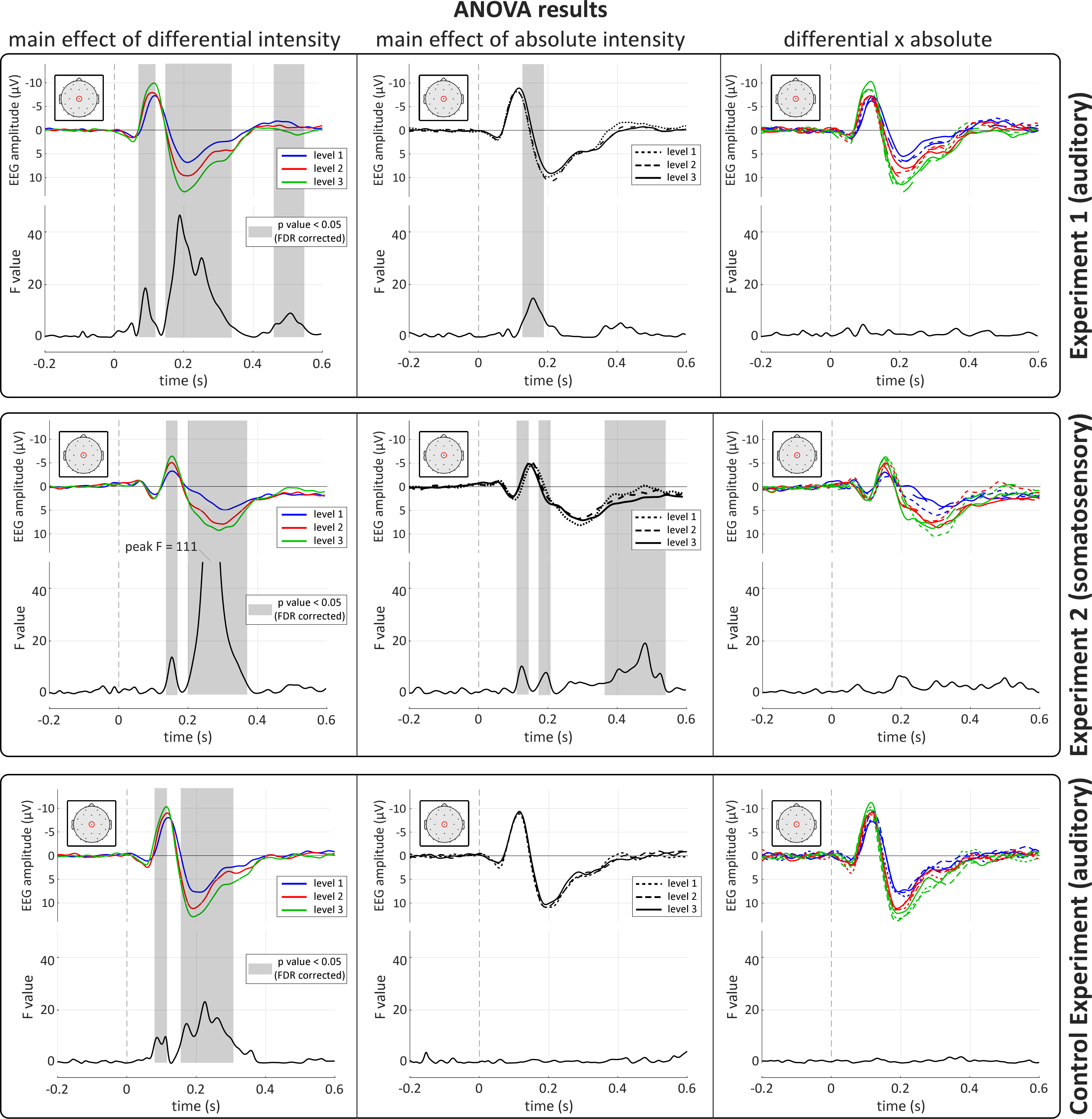

Supplement: figure_S2_bhaa267 [file figure_s2_bhaa267.png]
