# Supplementary material for: Waves of Change: Brain Sensitivity to Differential, not Absolute, Stimulus Intensity is Conserved Across Humans and Rats
Source: Cereb Cortex. 2020 Oct 7;31(2):949–60. doi: 10.1093/cercor/bhaa267 (PMC7786352; doi:10.1093/cercor/bhaa267)
Supplement: Somervail_et_al_supplementary_figure_legends_bhaa267 [file somervail_et_al_supplementary_figure_legends_bhaa267.docx]

[Somervail](javascript:popWindow('cercor?PARAMS=xik_HfxERro7Ax2eEDFufjAoHtSpTy8iYmozXn9vGnpLNiYKGR3RewaBUb4duioHmjARD8UWvaXAuF2jrzg9sDzE4jjbFJpo9Xvue9gEfPo839Rxvjkzh6WM3H9BGjVsvWXgYGxWJpfQtuUY8rhbxqKPNvxmLhiqfvXLKUHwBKqPMy9gbrG','mailpopup_1431',%20900,%20775);) et al supplementary figure legends

Figure S1. Control Experiment results.This additional experiment was performed in 8 participants (3 female, mean age 32, age range 25 - 45). Top panels. Results of point-by-point LME analysis. Top plots show group-level average waveforms at Cz for each of the three levels of differential (left panel) and absolute intensity (right panel). Bottom plots show the LME model coefficient timecourse for each factor. Grey areas show significant clusters from permutation testing. Vertical dashed lines indicate stimulus onset. Both negative and positive VPs were strongly modulated by the factor ‘differential intensity’. The peak topographies of these effects correspond well to those of the EEG response. There was no evidence of a modulation of the EEG by the factor ‘absolute intensity’. Bottom panel. Group level average waveforms at Cz, for each condition. Each row shows all 9 conditions of the experiment. Insets show schematic stimulus profiles, for each condition. Note the effect of differential, but not absolute intensity on both the negative and positive waves.

Figure S2. ANOVA results of Experiments 1, 2 and Control Experiment.Each row shows the results of a two-way repeated-measures ANOVA conducted on the subject-level average waveforms. There was no evidence for an interaction of the factors ‘differential intensity’ and ‘absolute intensity’ in any of the three experiments, whereas the main effects of these two factors are highly similar to those found in the main analysis using linear mixed-effect model.
